# Does vancomycin prescribing intervention affect vancomycin-resistant enterococcus infection and colonization in hospitals? A systematic review

**DOI:** 10.1186/1471-2334-7-24

**Published:** 2007-04-10

**Authors:** Monique A de Bruin, Lee W Riley

**Affiliations:** 1 Department of Medicine, Stanford University School of Medicine, Medicine Residency Office S101 (m/c 5109), Stanford, CA 94305, USA; 2 Divisions of Infectious Diseases and Epidemiology, School of Public Health, University of California, Berkeley 140 Warren Hall, Berkeley, CA, 94720, USA

## Abstract

**Background:**

Vancomycin resistant enterococcus (VRE) is a major cause of nosocomial infections in the United States and may be associated with greater morbidity, mortality, and healthcare costs than vancomycin-susceptible enterococcus. Current guidelines for the control of VRE include prudent use of vancomycin. While vancomycin exposure appears to be a risk factor for VRE acquisition in individual patients, the effect of vancomycin usage at the population level is not known. We conducted a systematic review to determine the impact of reducing vancomycin use through prescribing interventions on the prevalence and incidence of VRE colonization and infection in hospitals within the United States.

**Methods:**

To identify relevant studies, we searched three electronic databases, and hand searched selected journals. Thirteen studies from 12 articles met our inclusion criteria. Data were extracted and summarized for study setting, design, patient characteristics, types of intervention(s), and outcome measures. The relative risk, 95% confidence interval, and p-value associated with change in VRE acquisition pre- and post-vancomycin prescription interventions were calculated and compared. Heterogeneity in study results was formally explored by stratified analysis.

**Results:**

No randomized clinical trials on this topic were found. Each of the 13 included studies used a quasi-experimental design of low hierarchy. Seven of the 13 studies reported statistically significant reductions in VRE acquisition following interventions, three studies reported no significant change, and three studies reported increases in VRE acquisition, one of which reported statistical significance. Results ranged from a reduction of 82.5% to an increase of 475%. Studies of specific wards, which included sicker patients, were more likely to report positive results than studies of an entire hospital including general inpatients (Fisher's exact test 0.029). The type of intervention, endemicity status, type of study design, and the duration of intervention were not found to significantly modify the results. Among the six studies that implemented vancomycin reduction strategies as the sole intervention, two of six (33%) found a significant reduction in VRE colonization and/or infection. In contrast, among studies implementing additional VRE control measures, five of seven (71%) reported a significant reduction.

**Conclusion:**

It was not possible to conclusively determine a potential role for vancomycin usage reductions in controlling VRE colonization and infection in hospitals in the United States. The effectiveness of such interventions and their sustainability remains poorly defined because of the heterogeneity and quality of studies. Future research using high-quality study designs and implementing vancomycin as the sole intervention are needed to answer this question.

## Background

Since emerging 20 years ago [1], vancomycin-resistant enterococcus (VRE) has spread throughout the world to become a major cause of nosocomial infections.  American hospitals have experienced a particularly dramatic increase in the occurrence of VRE colonization and infection [2]. According to the Center for Disease Control and Prevention (CDC), the percentage of enterococcal isolates resistant to vancomycin reported by United States (US) hospitals increased from 0.3% in 1989 to over 25% of all isolates in 1999 [2]. Data from the 2004 National Nosocomial Infection Survey indicated that VRE caused approximately one third of infections in intensive care units (ICUs).

The reported increases in VRE colonization and infection among hospitalized patients are of concern for several reasons. Infections due to VRE may be associated with greater morbidity, mortality, lengths of stay and hospital costs than those due to vancomycin-susceptible enterococci (VSE), independent of co-morbid conditions that may have led to infection [3,4]. Of note, this topic remains controversial as some investigators have reported a lack of association between VRE infections and increased morbidity and mortality [5-11].

The progression of VRE colonization and infection at an institution is often from sporadic cases to monoclonal outbreaks and then to polyclonal endemicity. Once established, endemicity is difficult to eradicate [12-14]. Due to the combination of intrinsic and acquired resistance, treatment options for infections caused by VRE are extremely limited. Transfer of vancomycin resistance determinants to more virulent organisms is also of great concern, and vancomycin-intermediate and vancomycin-resistant *Staphylococcus aureus *have already emerged [15].

In 1995, as a response to rising rates of VRE colonization and infection, the CDC's Hospital Infection Control Practices Advisory Committee (HICPAC) established guidelines for control of VRE. These include prudent vancomycin use, as well as infection control measures to limit cross-contamination. Despite these recommendations, the precise association between vancomycin use and VRE colonization and infection remains unclear. Substantial research from animal models [1,16,17] and individual-level studies [18-20] has supported a role of vancomycin use in contributing to VRE acquisition. In a meta-analysis by Carmeli et al., among 15 individual-level studies using optimal control groups, vancomycin exposure conferred a 2.7-fold increased risk of VRE acquisition [21]. In addition to being a risk factor for VRE acquisition, vancomycin exposure may also increase VRE detection in patients already colonized, by eliminating other colonizing bacteria and allowing VRE to flourish [22].

There are conflicting data, however, that suggest that antecedent vancomycin exposure may not pose significant risk to individual patients [22-24]. In fact, a review by Harbarth et al. on antibiotic exposure and VRE concluded that intravenous vancomycin use may have a limited role in contributing to new VRE acquisition, while cephalosporins and anti-anaerobic agents may have a greater effect.

Even less clear is the role of vancomycin restriction and control on clinically significant VRE acquisition at the population level. Data from the largest ecologic study to date showed that vancomycin was the most significant 'modifiable' risk factor leading to VRE colonization [25]. Yet results from studies investigating the impact of reductions in vancomycin use on VRE colonization and infection have been heterogeneous [20,26-36]. We conducted a systematic review of the available literature to evaluate the impact of reducing vancomycin use through vancomycin prescribing interventions on the prevalence and incidence of VRE colonization and infection in US hospitals.

## Methods

### Search strategy

We searched the following electronic databases: PubMed including Medline, as well as Web of Science, and the Cochrane Library. Searches were limited to studies published from 1987 (the year VRE was first reported in the US) to March 2006 (the date the search was conducted); searches were also limited to human subjects. The search terms included "*Enterococcus*," "*Enterococcus faecalis*," "*Enterococcus faecium*," "vancomycin," "antibiotic resistance," "antimicrobial resistance," "drug resistance," "prescribing," "guidelines," "restriction," "colonization," and "infection." All titles and abstracts (if available) from each of the searches were examined, with relevant articles being obtained for review. In articles with unclear relevance, full text versions were also scanned. Subsequently, reference lists of all identified reports, studies and reviews were screened to identify additional studies. Although we did not impose language restriction while searching, we included only English language articles in our review.

### Selection criteria

Inclusion criteria were established before identifying articles to avoid selection bias. Eligible studies were experimental in design (randomized controlled trials or quasi-experimental studies), conducted within US hospitals, and described the impact of significant reductions in vancomycin use, following any intervention(s) aiming to reduce its use, on the prevalence or incidence of VRE. Measurements of VRE acquisition (colonization or infection) within a control group not exposed to the intervention were required for comparison, although no minimum number of data points was imposed. Only published studies were included.

We excluded the following: studies outside of the United States (since the epidemiology of VRE is different elsewhere), animal studies, reviews, case reports and observational studies. Also excluded were studies that did not observe a significant reduction in vancomycin use following an intervention aimed at doing so since this was the intervention of interest. Finally, we excluded studies with a focus on antecedent vancomycin use as a risk factor for VRE acquisition in individual patients, as this review focused on population level associations. The review could not be limited to studies investigating the effect of vancomycin use reductions alone on VRE colonization and infection, as several studies simultaneously investigated other interventions, such as restriction of other antibiotics and infection control measures. If a trial had multiple published reports, the latest reports, with the longest duration of follow up, were included. No studies were excluded based on quality criteria alone.

### Data extraction and outcome measures

The final set of included articles was assessed by one reviewer (MAD), who extracted data from all studies. The reviewer was not blinded to the names of the authors, institutions, journal of publication or study results. Information was collected on the publication year, study period, setting, design, number of participants, selection and characteristics of participants, laboratory methods for determination of VRE infection status, characteristics and timing of interventions, duration of follow-up, timing and frequency of outcome measurements, and the reporting of other possible confounding variables.

The outcome measures of interest were extracted from each study. These included measurements of vancomycin use and prevalence or incidence of VRE acquisition before and after an intervention to reduce vancomycin usage, and the corresponding confidence intervals (95%) when provided. Data from the entire pre- and post-intervention periods, or as much as reported, were included. When several months or years of pre- and post-intervention data were reported separately, the data for each period was combined. For example, data reported as the incidence of cases in each individual month were combined to find the cases per day over all months in the time period, assuming 30 days per month. For studies in which an intervention took place during a certain month, the prior month(s) were included as pre-intervention and following month(s) as post-intervention, but the actual month of intervention was excluded from these periods.

### Data analysis and statistical methods

Absolute and relative (percentage) changes in vancomycin use and VRE acquisition were calculated from pre- and post-intervention data. The relative risk (RR) of VRE colonization or infection in the intervention group compared with the control group was calculated by dividing the probability of VRE acquisition in the post-intervention period by the probability of VRE acquisition in the pre-intervention period. A RR below one represented a protective effect of vancomycin reduction on VRE colonization or infection. When sufficient data were reported, the 95% confidence interval for the RR and the p-value were calculated by EpiInfo StatCalc. Statistical significance was indicated by a p-value of less than 0.05. For studies reporting cases per unit of time without including the number of patients tested, it was assumed that the number of patients tested per unit of time was constant. When sample size was reported in terms of the number of cultures over the entire study period, it was assumed that a constant number of cultures were obtained for each unit of time, in order to obtain the sample size for pre- and post-intervention periods, and thereby permit calculation of the confidence intervals for relative risk.

Studies were categorized according to the hierarchy of quasi-experimental study designs [37]. According to this classification scheme, category A studies do not use control groups, while category B studies do. A1 studies use a 1-group pretest-posttest design and A2 studies use a 1-group pretest-posttest design with a double pretest. We have chosen to denote A1 studies which use multiple posttest measurements as "A1*" and A2 studies using multiple posttest measurements as "A2^†^." In general, studies using a control group are of higher quality than those without controls, and studies with multiple pretest and posttest measurements are preferable to those without such repeated measurements. For a more complete review on the subject, see bibliographical reference 36.

Because of the heterogeneity among study designs, populations, interventions, and quality, a pooled summary estimate would not provide a meaningful estimate of effect; therefore, meta-analysis was not performed. Instead, a simple vote count was tabulated to determine the number of studies finding a significant decrease, increase or no change in prevalence or incidence of VRE colonization and/or infection. The range of point estimates for each category of studies was noted.

To explore heterogeneity, we stratified studies by pre-specified covariates, each having clinical rationale, to investigate possible effect modification. Covariates included 1) vancomycin reduction alone vs. concomitant implementation of other VRE control interventions, 2) ward-specific vs. hospital-wide interventions, 3) severity of illness of the study population, 4) outbreak status of the institution or ward(s) under investigation, 5) type of quasi-experimental design and 6) duration of intervention.

The proportion of studies in each stratum finding an increase, decrease and no change in VRE colonization and/or infection, and the range of point estimates within each stratum was noted. The quality of each study was assessed by informal methodological criteria, and possible sources of bias were investigated. Discrepancies in study quality were examined as potential contributors to inter-study heterogeneity.

## Results

### Search results

Of the 2027 citations identified after literature searches, 12 were judged to meet our inclusion criteria and were included in this review [20,26-36]. All included studies presented pre- and post-intervention data, with the prevalence or incidence of VRE colonization and/or infection compared before and after implementation of a vancomycin use reduction intervention at the institution or ward under investigation. No similar systematic reviews or randomized controlled trials were found.

### Description of included studies

Table 1 describes the major characteristics of each study included in the review. A total of 12 articles describing 13 studies are included. The studies were published between 1992 and 2005. They were clustered around the lowest ranking designs of the classification of quasi-experimental studies proposed by Harris et al. [37] (Table 1). All studies lacked a control group and none removed and reintroduced interventions. Eleven of 13 took place at a single teaching hospital [20,26-29,31-33,35,36], one was at a non-teaching community hospital [30] and one was a multi-center trial of both academic and community hospitals [34]. One study reported initial cases of VRE colonization [26], two reported current outbreaks without an endemic problem [27,28], and one reported a superimposed outbreak on an endemic problem with VRE infections [31]. Aside from the multi-site study that did not specify outbreak status [34], the remaining studies described a VRE problem of several years duration [20,26,29,30,32,33,35,36]. No article provided explicit definitions for outbreak vs. endemicity, and classifications may have varied. One study reporting an outbreak described it as lasting over four years [27]; it is possible that other studies may have considered this type of time frame to constitute endemicity.

**Table 1:** Characteristics of studies included in the systematic review

**Study**	**Setting**	**Study population**	**VRE at institution**	**Study design**	**Intervention(s)**	**Length of follow up**	**Vancomycin use outcome**	**VRE outcome**
[26]	Children's hospital	Pediatric oncology and ICU	Initial cases	A2^†^	VR + IC	5 mos.	Proportion of patients receiving	Prevalence of colonization
[20]	Academic hospital	General inpatient	Endemic	A2^†^	IC → VR	6 mos.	PO and IV doses/mo.	Prevalence of colonization
[27]	VA hospital	General inpatient	Outbreak	1)A1 2)A2^†^	IC→ restrict vanco, ceftazidime and clindamycin	6 mos.	PO and IV units/mo.	Prevalence of colonization, incidence of positive clinical isolates
[28]	Academic hospital	"High risk ward"	Outbreak	A1*	VR + IC	4 mos.	% of orders inappropriate per HICPAC, grams/1,000 pt-days	Incidence of positive surveillance cultures
[29]	Academic hospital	General inpatient	Endemic	A1	VR	24 mos.	Grams/1,000 pt-days, total # of patients exposed	Incidence as % of all enterococcal isolates resistant to vanco
[30]	Non-teaching community hospital	General inpatient	Endemic	A2^†^	Vanco education program → VR	24 mos.	Doses purchased/1,000 pt-days, dollar purchases/1,000 pt-days, empiric treatment, treatment with positive culture	Incidence of colonization or infection (quarterly)
[31]	ICUs of an academic hospital	ICU	Endemic with super-imposed outbreaks	A2^†^	IC + progressive VR	19 mos.	Doses/1,000 pt-days	Incidence of colonization and infection (monthly)
[32]	Academic oncology ward	Oncology	Endemic	A1	IC→ enhanced IC (included efforts to reduce use of all antimicrobials) + VR	12 mos.	Grams/1,000 pt-days	Incidence of colonization/1,000 pt-days and BSI/1,000 pt-days
[33]	Academic cancer center's BMT and leukemia ward	Heme malignancy	Endemic	A1*	VR + IC	36 mos.	Empiric use in grams/1,000 pt-days Cost/1,000 pt-days	Incidence of total infections/1,000 pt-days and BSI/1,000 pt-days
[34]	50 ICU's at 20 hospitals; academic, VA and community	ICU	Not reported	A1	Vanco use local monitoring data compared with national benchmarks	23 mos.	DDD/1,000 pt-days	Prevalence of positive clinical isolates
[35]	Academic hospital	General inpatient	Endemic	A1*	Progressive VR	4 yrs.	DDD/1,000 pt-days	Prevalence as annual % of enterococcal isolates resistant to vanco
[36]	Academic hospital	General inpatient	Endemic	A1	VR	8 yrs	% of orders inappropriate per HICPAC guidelines; grams/1,000 pt-days	Incidence: infections/yr
[36]	ICU	ICU	Endemic	A1*	Multidisciplinary ICU team to reduce vanco use	4 yrs.	% of orders inappropriate per HICPAC guidelines	Incidence: infections/yr, infections/1,000 pt-days

Abbreviations: ICU = intensive care unit, BMT = bone marrow transplant, VA = Veteran's Affairs, → = followed by, DDD = defined daily dose, HICPAC = Hospital Infection Control Practices Advisory Committee, Pt-days = patient-days, BSI = blood stream infections, VR = vancomycin restriction, IC = infection control, vanco = vancomycin

Footnotes: A1 = 1-group pretest-posttest design; A2 = 1-group pretest-posttest design that uses a double pre-test (for a complete description of hierarchy of quasi-experimental design studies, see Harris et al.), * Category A1 with multiple post-tests;^† ^Category A2 with multiple post-tests

Six studies implemented institution-wide interventions to a population of general inpatients [20,27,29,30,35,36], while seven performed ward-specific interventions including malignant hematology [33], oncology [26,32], ICU [26,31,34,36] or "high-risk" [28] patients. For all studies, the comparison group consisted of patients in the same hospital or ward as the intervention group, but prior to the implementation of the intervention. Therefore, the two groups differed temporally. Few studies provided comparison of key features of the two groups, including type and severity of illness, length of stay, and other features known to affect the risk of VRE acquisition. The types of interventions, and the timing and sequence of implementation varied widely. Six studies implemented vancomycin reduction efforts alone [29,30,34-36], five implemented vancomycin reduction efforts plus infection control [20,26,28,31,33], and the remaining studies used a combination of vancomycin reduction efforts, infection control efforts, and efforts to reduce the use of other classes of antibiotics [27,32,35]. All studies conducted during outbreaks [27,28,31] involved multiple interventions simultaneously, presumably due to the urgency of controlling the outbreak. Studies used several different measures and units for tracking changes in vancomycin use.

Measures of VRE included colonization and/or infection, with studies varying on which they used, whether they provided definitions for their measure(s), and if provided, which definitions they used. Two studies reported measuring colonization only [20,26]. One study reported measuring the percentage of VRE in surveillance cultures of high risk patients, and it is unclear whether this number represents colonization with or without infection [28]. Three studies reported the proportion of enterococcal isolates resistant to vancomycin among specimens ordered for clinical reasons [29,34,35], and the study by Morgan et al. states explicitly that this number represents both colonization and infection [29]. Three studies reported colonization and infection separately [27,32,33]. All definitions of colonization were similar, defined as the proportion of surveillance cultures or proportion of a point prevalence survey that were VRE-positive. Three studies reported infections without providing definitions [30,36] and two stated using the CDC definition of infection [32,33].

Studies used various sampling methods to detect cases of VRE acquisition, including testing all patients in a study population at a given time [26,33], obtaining random samples of study participants and screening for VRE [20,27], sampling patients thought to be at high risk of VRE acquisition [28,31], and obtaining clinical isolates [29,30,34-36]. As such, sample size varied from a relatively small sample of the inpatient census at a given time to the entire patient census at a given time, to all patients admitted to a hospital over several years. Measures of disease occurrence included serial point prevalence surveys or determination of incidence, which involved evaluating the number of new cases over time or patient-days. The duration of follow up ranged from four months to eight years, with a median duration of 23 months and a mean duration of 27 months. Little or no data were reported on the following characteristics of study participants: age, gender, or underlying illnesses including HIV.

### Overall impact of vancomycin reductions on VRE acquisition

Table 2 describes the relative reduction in vancomycin use and changes in VRE acquisition for each of the studies. All articles, by definition of inclusion, reported a significant reduction in vancomycin use. The study by Lai et al. [31] reports initial unsuccessful attempts to reduce vancomycin use, followed by vancomycin restriction efforts which were successful. Our analysis of this study includes only the portion in which vancomycin was restricted, during which time vancomycin usage was significantly reduced. The study by Lautenbach et al. [35] reports an initial significant reduction in vancomycin usage, but then usage returned to pre-intervention levels by the end of the study. In this case, we included only the initial period during which vancomycin use was significantly reduced, as the latter portion did not meet inclusion criteria. The multi-center trial by Fridkin et al. describes reductions in vancomycin use for sites implementing unit-specific interventions but not for those implementing hospital-wide interventions; only data from sites with unit-specific interventions were included [34]. The article by Guglielmo et al. includes a hospital-wide intervention to reduce vancomycin use and corresponding changes in VRE infections, as well as a later ICU-specific intervention, again with corresponding changes in VRE infections [36]. Since the results of these two interventions are reported separately in their article, they are discussed as separate studies in this review, and count as separate votes in the stratified analysis.

**Table 2:** Study results – reductions in vancomycin use and changes in VRE

**Reference**	**Vancomycin use % reduction**	**VRE acquisition absolute change**	**VRE acquisition percentage change**	**Significant change in VRE acquisition?**	**RR of VRE after intervention(s)** **(95% CI)**
Rubin et al [26], 1992	66%	-.157 proportion of patients colonized (prevalence)	-82.5%	p = 0.023 (FET)	0.17 (0.04, 0.72)
Morris et al [20], 1995	66%	-0% mean rate of stool colonization (prevalence)	0%	NS	0.96 (0.59, 1.57)
Quale et al [27], 1996^a^	44%	-32% point prevalence of fecal colonization -2.1 new patients with positive cultures per month (incidence)	-68% (prevalence) -39% (incidence)	p < 0.001 (prevalence) p = 0.03 (incidence)	0.32 (0.22, 0.46) (prevalence) * (incidence)
Anglim et al [28], 1997	47%	-0.52 cases/day (incidence)	-52.2%	Chi-square test for trend, 16.6; p = 0.002	0.48 (0.31, 0.75)
Morgan et al [29], 1997	9%	-0% of all enterococcal isolates resistant to vancomycin (incidence)	0%	NS	1 (*)
Adachi et al [30], 1997	54%	"No further increase" (incidence)	~0%	NS	1.75 (*)
Lai et al [31], 1998^b^	25%	+2.46 cases/month (incidence)	+33.5%	*	1.33 (*)
Montecalvo et al [42], 1999	28%	-10.4 pts/1,000 pt-days (colonization) -1.65 pts/1,000 pt-days (BSI) (incidence)	-50% (colonization) -79% (BSI)	p < 0.001 (colonization) p = 0.04 (BSI)	0.5 (0.33, 0.75) (colonization) 0.22 (0.05, 0.92) (BSI)
Shaikh et al [33], 2002	50% (g/1,000 pt-days) 53% ($/1,000 pt-days)	-0.21 cases/1,000 pt-days (total incidence) -0.157/1,000 pt-days (BSI) (incidence)	-48% (total incidence) -46% (BSI)	p = 0.008 (total infections) p = 0.027 (BSI)	0.52* (total incidence) 0.54* (BSI)
Fridkin et al [34], 2002^c^	35–37% median difference	-7.5% mean difference in prevalence	Unable to calculate with provided data	"Statistical significance" *	*
Lautenbach et al. [35], 2001^d^	Period 3 vs. 1: 26.3% Period 2 vs. 1: 8.1% Period 3 vs. 2: 19.8%	% of enterococcal isolates resistant to vancomycin (prevalence): Period 3 vs. 1: +9.5% Period 2 vs. 1: +7.6% Period 3 vs. 2: +1.9%	Period 3 vs. 1: +54.6% Period 2 vs. 1: +43.7% Period 3 vs. 2: +7.6%	p < 0.001 (detected with χ^2 ^test for trend)	Period 3 vs. 1: 1.55 (1.37, 1.74) Period 2 vs. 1: 1.44 (1.27, 1.62) Period 3 vs. 2: 1.076 (0.968, 1.195)
Guglielmo et al. [36] 2005 (hospital-wide study)	86.7% inappropriate use	+7.94 infections/month (incidence)	475%	*	5.77 (*)
Guglielmo et al. [36] (ICU-specific study)	33.4% inappropriate use	-1.17 infections/month -4.1 infections/1,000 pt days (incidence)	-48% (infections/month), -65.1% (infections/1,000 pt days)	p = 0.0003 (infections/1,000pt-days)	0.517 (infections/month), 0.35 (0.22, 0.57) (infections/1,000 pt days)

Abbreviations: FET = Fisher's exact test, NS = not significant, BSI = blood stream infection

*Insufficient data reported to perform calculation

^a^Quale: only prevalence data used to calculate significance, RR (CI); insufficient data to calculate for incidence

^b^Lai: data on VRE incidence are from five months prior to intervention and final five months of follow up

^c^Fridkin: includes only data from ICUs implementing unit-specific change

^d^Lautenbach: table includes data from initial three of four study periods; following are data from the fourth period compared with the first: *increase *in vancomycin use of 15.5%, VRE absolute change +6% of enterococcal isolates resistant to vancomycin, VRE acquisition % change +66.7%, significant increase with p < 0.001, RR = 1.70 (1.53, 1.89)

Overall, the 13 studies reported inconsistent findings. Seven studies [26-28], [32-34,36] reported statistically significant improvement in the prevalence or incidence of VRE colonization and/or infection, with relative reductions ranging from 46% [33] to 82.5% [26]. Three studies reported no change in levels of VRE colonization [20,30] or clinical isolates [29]. Three studies reported an increase in VRE measures, one of which was statistically significant [35]. The other two noted increases of 33.5% [31] and 475% [36], but with no indication of significance and with insufficient data reported to calculate the p-value.

### Stratified analysis

As described above, substantial heterogeneity was present in the direction and magnitude of study outcomes, likely reflecting the widely varying study settings, populations, and interventions. To explore this heterogeneity, we stratified by pre-specified covariates suspected of affecting the study results, and performed simple vote counts of the number of studies within each strata (Table 3).

**Table 3:** Stratification by covariates

**Variable**	**Number of studies**	**Significant % reduction in VRE acquisition**	**Fisher's exact test comparing strata**
Type of intervention			NS^1^
Vancomycin reduction alone	6	2	
Vancomycin plus other interventions	7	5	
Site-based^2^			(p = 0.029, FET^2^)
Ward alone	7	6	
Hospital-wide	6	1	
VRE acquisition occurrence^3^			NS
Outbreak only	2	2	
Endemic (with or without outbreak)	9	3	
Study design			NS
A1	5	3	
A1*	4	4	
A2 ^†^	5	2	
Duration of intervention			NS
≤ 6 months	4	3	
> 6 months	9	4	

^1 ^NS = not significant result of Fisher's exact test comparing strata

^2 ^Stratification by severity of illness yields identical results to the site-based stratification: "ward alone" and "high severity of illness" are identical strata; "hospital wide" and moderate severity of illness" are identical strata

^3^Excluded Rubin et al. (initial cases) and Fridkin et al. (multi-cite, outbreak/endemic status not reported)

A1 = 1-group pretest-posttest design; A2 = 1-group pretest-posttest design that uses a double pre-test (for a complete description of hierarchy of quasi-experimental design studies, see Harris et al.), * Category A1 with multiple post-tests; ^† ^Category A2 with multiple post-tests

Six studies (46%) implemented vancomycin reduction measures as the sole type of VRE control intervention [29,30,34-36]. The remaining seven studies also implemented infection control and/or restriction of additional antimicrobial agents [20,26-28,31-33]. Although it was not statistically significant, studies that controlled vancomycin alone revealed a trend towards lower efficacy in reducing VRE colonization and infection (33%) when compared to those that used additional measures (71%). Additionally, both studies (100%) restricting multiple classes of antimicrobial agents reported improvements [27,32].

We investigated whether results of studies varied by ward-specific vs. hospital-wide interventions and by severely ill vs. moderately ill patient populations. Both stratifications yielded identical results, as all ward-specific units included sicker patients. Six of seven studies (86%) focusing on ward-specific interventions and including severely ill patients reported significant reductions in VRE colonization and/or infection [26,28,32-34,36]; only one did not [31]. Of the studies implementing hospital-wide VRE control efforts and including moderately ill patients, only one [27] of the six [20,27,29,30,35,36] (17%) reported a reduction in VRE colonization or infection during the post-intervention period. Fisher's exact test comparing the strata yielded a p-value of 0.029. Two of the articles describe implementation of both unit-specific and hospital-wide interventions, and assessed the impact of each on VRE colonization and/or infection separately [34,36]. Both of these (100%) reported reductions in VRE infections for the ICU-specific interventions, but worsening of VRE infections for the hospital-wide interventions, with one also failing to reduce vancomycin use at the hospital level. This example may highlight the relative success of interventions implemented at the ward-level compared with the hospital-level.

Study results were suspected of varying by whether an institution was facing an outbreak and/or endemic problem with VRE. Outbreaks tend to be easier to control than endemic problems. Further, by definition, outbreaks are recognized as such when they are controlled [12,13,38]. All three studies (100%) at institutions without endemic VRE reported reductions in post-intervention VRE acquisition; two of these were amidst an outbreak [27,28] and one during the institution's initial cases [26]. The studies with an endemic VRE problem reported less favorable results: of the nine [20,26,29-33,35,36], only three (33%) [32,33,36] reported significant reductions in VRE colonization or infection and another three (33%) reported no improvement [20,29,30]. The study with an outbreak superimposed on an endemic VRE problem reported worsening of VRE acquisition [31], perhaps suggesting less success of interventions during an endemic problem, whether or not super-imposed outbreaks are present. As noted above, the multi-site study by Fridkin et al. was excluded from this analysis because it lacked information regarding outbreak status of the various sites [34].

To assess for an association between study design and results, we stratified studies according to the hierarchy of quasi-experimental study designs scheme [37]. Five studies were of 1-group pretest-posttest design (A1), four were of this same design but with multiple posttests (A1*), and five performed a double pretest with multiple posttests (A2^†^). No significant difference in outcomes based on the present small variations in design could be detected, perhaps due to the small number of studies in each category. For A1 classification, the proportion of studies finding a significant reduction in VRE was 3/5 (60%), for A1* studies, 4/4 (100%), and for A2^†^studies, 2/5 (40%) (see Table 1 footnote).

Finally, the duration of an intervention was assessed for its impact on results. Interventions may take time to have an impact on VRE acquisition, and in turn the efficacy of interventions may wane over time, particularly since there is a behavioral component to the types of interventions employed by studies. Furthermore, the background VRE epidemiology in the US is one of continued increase, so studies looking over a longer time period may have greater difficulty in achieving reductions in VRE. Three [26-28] of four studies [20,26-28] (75%) with interventions of less than or equal to six months reported significant improvement in VRE acquisition. Of note, these four studies were the earliest performed of all included in this review. Fewer studies with interventions lasting greater than six months saw improvement; four [32-34,36] of nine [29-36] (44%) reported significant reductions in VRE acquisition. Further, one of these studies [35] with interventions lasting seven years, reported that after four years, the interventions were no longer effective and vancomycin usage returned to baseline. Meanwhile, VRE acquisition increased throughout the study period.

## Discussion

Many US hospitals continue to struggle with worsening rates of VRE colonization and infection, and current guidelines for prudent vancomycin use are lacking in evidence for their effectiveness [39]. Determining the effect of this particular VRE control strategy would assist hospitals to better direct their infection control efforts. This review summarizes the impact of reductions in vancomycin use on VRE colonization and infection at the population level, based on a systematic review of the literature.

### Principal findings

Our findings indicate that a slight majority of studies demonstrated improvement in control of the organism following reductions in vancomycin use, although individual studies reported a wide range of results. Seven of thirteen studies (54%) reported reductions in VRE colonization and/or infection; three (23%) reported no significant change, and three (23%) reported increases. Overall the percentage change in VRE acquisition ranged from a reduction of 82.5% [26] to an increase of 475% [36]. Interestingly, of six studies that used vancomycin restriction as the sole intervention, only two showed a significant reduction in VRE acquisition, whereas of the seven that implemented antimicrobial drug restriction plus infection control, five showed a significant VRE acquisition reduction. This observation suggests that an effective intervention for VRE infection and colonization control may require more than drug restriction alone.

### Limitations of this review

Our analysis has significant limitations related to the methodology and reporting of the available studies. None of the 13 studies reviewed in this report used experimental designs that would have allowed a definitive answer to our main question. Each of these studies used a quasi-experimental approach of lower hierarchy. In fact, all the studies fell below category A3 of the classification proposed by Harris et al. [37] (Table 1). Quasi-experimental studies possess significant limitations including 1) difficulty in controlling for important confounding variables (due to lack of randomization), 2) results that are explained by the statistical principle of regression to the mean (the principle that elevated rates will tend to return to baseline, even without an intervention) and 3) maturation effects (natural changes over time) [40].

None of these studies used segmented regression analysis nor were they randomized clinical trials. To evaluate the efficacy of a single intervention (e.g. reduction in vancomycin usage), randomized clinical trials may be ethically unacceptable given that commonly there is urgency in controlling an infectious agent or disease (e.g. VRE colonization and infection) in a timely manner and in including other interventions (e.g. infection control, reduction in the usage of other antibiotics). An alternative to randomized clinical trials may be the use of segmented regression analysis. This experimental approach is considered superior in design to other types of quasi-experimental studies by several investigators [33]. Segmented regression analysis enhances the internal validity of results, as compared with a simple comparison of composite data from pre- and post-intervention periods. None of the 13 studies obtained or provided sufficient data for us to perform a segmented regression analysis. To perform such analysis would have required collection of pre- and post-intervention data at equally spaced time intervals that span enough periods to detect pre-existing trends and cyclical patterns. Specifically, according to Wagner et al. [41], a general recommendation is to obtain 12 data points both before and after the intervention.

Most studies provided no data comparing pre- and post-intervention groups, which would be helpful since studies are not randomized. All but two studies were conducted at a single institution, limiting the generalizeability of results. Most studies did not adjust vancomycin usage rates for patient-days at risk. Morgan et al. demonstrated the importance of this adjustment [29]. Based on unadjusted data, they found a significant reduction in vancomycin usage; however, after adjusting for total patient days and for the percentage of patients receiving vancomycin, the two groups appeared to be "similar" in terms of vancomycin usage (no p-value reported). Often concurrent interventions were implemented, which likely confounded the results. Studies did not assess the influence of "contact patterns" (i.e. individual contact episodes with VRE carriers) and "colonization pressure" (the overall proportion of patients colonized with VRE in the unit), which are central components of VRE transmission, since the organism is predominantly spread by cross-contamination.

The heterogeneity of studies and results were of concern, and prevented us from conducting meta-analysis. Simple vote counts of individual studies were instead performed; these methods are crude and do not take into account sample size or variance, and do not provide a summary estimate of effect. In performing subgroup analysis by key variables to explore heterogeneity, the number of studies in individual strata was small. Therefore, our ability to detect a significant difference may have been limited by these small numbers.

Based on sub-group analysis by simple vote counts, results appeared to vary by the following covariates: type of intervention, ward-specific vs. hospital-wide interventions (significant p-value), severity of illness of patient populations (significant p-value), VRE acquisition occurrence at the institution, study design, and the duration of the intervention. Studies with longer durations of interventions often reported less successful results. As an example, Lautenbach et al. [35] report that after the fourth year of vancomycin reduction efforts, vancomycin usage had increased to baseline, while VRE acquisition continued to increase throughout the study period. Such findings may relate to challenges with sustaining interventions, as well as background increases in the overall prevalence and incidence of VRE within the United States.

Other possible explanations for the observed heterogeneity include variability in study quality, interventions to reduce vancomycin, study populations and their selection, differences between interventions and control groups including non-concurrence, differences in outcome measures and duration of follow up. For  example, the setting and design of studies evolved over the 13 years.  Earlier  studies involved initial cases of VRE or outbreaks, and follow up lasted only  months; in contrast, recent studies were in endemic settings and follow up  lasted up to a decade. Lack of control for confounding variables may also be affecting study results differentially.

In addition to heterogeneity across studies and the presence of confounding variables, other possible reasons for the mixed results should be considered. In some studies, vancomycin usage rates may have been insufficiently reduced to realize benefit or cross-contamination may have limited the efficacy of vancomycin reduction interventions. Alternatively, targeting antibiotic use, including vancomycin, may not be sufficient to reduce the incidence of VRE cases in endemic settings, since a significant number of infected or colonized patients may continue to serve as reservoirs.

### Suggestions for the design of future studies

Several of the methodological shortcomings observed in the studies reviewed here could be improved upon in future research. Ideally, randomized clinical trials should be performed. However, this design is often considered impractical by infection control personnel and may be unethical in outbreak settings. Alternatively, implementing higher quality quasi-experimental study designs (e.g. category B designs, which use control groups) or using segmented regression analysis may allow for more causal interpretation of observed associations than using quasi-experimental designs of lower quality (e.g. less than A3) [37,40]. When possible, distinct control groups should be used. Collection of additional data points before and after implementation of an intervention may shed further light on baseline trends, the immediate and sustained impacts of the intervention, maturation effect, and cyclical/seasonal patterns. By having sufficient data points to perform segmented regression analysis, the internal validity of studies would be improved.

In order to clearly answer the question of whether reduction in vancomycin usage results in a decrease of VRE colonization and disease, a significant decrease in vancomycin prescriptions should be the only variable introduced. This approach would not be possible in outbreak settings. However, it would be feasible in endemic settings where there is less of a need for concurrent implementation of infection control measures. Additionally, collection of adequate pre-intervention data points would be more possible because of relatively less urgency. More discussion of why authors chose their particular study designs, as well as their strengths and limitations would be helpful to readers and in the design of future studies. Standardized nomenclature regarding study designs should be implemented to enhance the clarity of research designs and methodology.

## Conclusion

In summary, based on this systematic review of the literature, it was not possible to conclusively determine a potential role for vancomycin usage reductions in controlling VRE colonization and infection in hospitals in the Untied States, as is recommended by current guidelines [39]. The effectiveness of such interventions and their sustainability remain poorly defined because of heterogeneity in study design and results, as well as insufficient study quality to enable adequate causal inference. In general, studies implementing vancomycin reduction as the sole intervention to control VRE were less successful than those implementing additional VRE control strategies, although there may have been too few studies to detect a significant difference between groups. Future research using experimental designs of higher quality and implementing vancomycin use reduction as the sole intervention is needed to answer this question.

## Competing interests

The author(s) declare that they have no competing interests.

## Authors' contributions

MAD designed the study, searched the databases, extracted the data, analyzed the results and wrote the manuscript. LWR conceived of the study, participated in its design and coordination, assisted with analysis and interpretation of data, and provided critical revision of the manuscript for important intellectual content. Both authors read and approved the final manuscript.

## Pre-publication history

The pre-publication history for this paper can be accessed here:



## References

[B1] Leclercq R, Derlot E, Duval J, Courvalin P (1988). Plasmid-mediated resistance to vancomycin and teicoplanin in Enterococcus faecium. N Engl J Med.

[B2] (2000). National Nosocomial Infections Surveillance (NNIS) system report, data summary from January 1992-April 2000, issued June 2000. Am J Infect Control.

[B3] Stosor V, Peterson LR, Postelnick M, Noskin GA (1998). Enterococcus faecium bacteremia: does vancomycin resistance make a difference?. Arch Intern Med.

[B4] Salgado CD, Farr BM (2003). Outcomes associated with vancomycin-resistant enterococci: a meta-analysis. Infect Control Hosp Epidemiol.

[B5] DiazGranados CA, Zimmer SM, Klein M, Jernigan JA (2005). Comparison of mortality associated with vancomycin-resistant and vancomycin-susceptible enterococcal bloodstream infections: a meta-analysis. Clin Infect Dis.

[B6] Lautenbach E, Bilker WB, Brennan PJ (1999). Enterococcal bacteremia: risk factors for vancomycin resistance and predictors of mortality. Infect Control Hosp Epidemiol.

[B7] Stroud L, Edwards J, Danzing L, Culver D, Gaynes R (1996). Risk factors for mortality associated with enterococcal bloodstream infections. Infect Control Hosp Epidemiol.

[B8] Garbutt JM, Ventrapragada M, Littenberg B, Mundy LM (2000). Association between resistance to vancomycin and death in cases of Enterococcus faecium bacteremia. Clin Infect Dis.

[B9] Shay DK, Maloney SA, Montecalvo M, Banerjee S, Wormser GP, Arduino MJ, Bland LA, Jarvis WR (1995). Epidemiology and mortality risk of vancomycin-resistant enterococcal bloodstream infections. J Infect Dis.

[B10] Lucas GM, Lechtzin N, Puryear DW, Yau LL, Flexner CW, Moore RD (1998). Vancomycin-resistant and vancomycin-susceptible enterococcal bacteremia: comparison of clinical features and outcomes. Clin Infect Dis.

[B11] Peset V, Tallon P, Sola C, Sanchez E, Sarrion A, Perez-Belles C, Vindel A, Canton E, Gobernado M (2000). Epidemiological, microbiological, clinical, and prognostic factors of bacteremia caused by high-level vancomycin-resistant Enterococcus species. Eur J Clin Microbiol Infect Dis.

[B12] Kim WJ, Weinstein RA, Hayden MK (1999). The changing molecular epidemiology and establishment of endemicity of vancomycin resistance in enterococci at one hospital over a 6-year period. J Infect Dis.

[B13] Stosor V, Kruszynski J, Suriano T, Noskin GA, Peterson LR (1999). Molecular epidemiology of vancomycin-resistant enterococci: a 2-year perspective. Infect Control Hosp Epidemiol.

[B14] Nelson RR, McGregor KF, Brown AR, Amyes SG, Young H (2000). Isolation and characterization of glycopeptide-resistant enterococci from hospitalized patients over a 30-month period. J Clin Microbiol.

[B15] Appelbaum PC (2006). The emergence of vancomycin-intermediate and vancomycin-resistant Staphylococcus aureus. Clin Microbiol Infect.

[B16] Wong MT, Kauffman CA, Standiford HC, Linden P, Fort G, Fuchs HJ, Porter SB, Wenzel RP (2001). Effective suppression of vancomycin-resistant Enterococcus species in asymptomatic gastrointestinal carriers by a novel glycolipodepsipeptide, ramoplanin. Clin Infect Dis.

[B17] Pultz NJ, Stiefel U, Donskey CJ (2005). Effects of daptomycin, linezolid, and vancomycin on establishment of intestinal colonization with vancomycin-resistant enterococci and extended-spectrum-beta-lactamase-producing Klebsiella pneumoniae in mice. Antimicrob Agents Chemother.

[B18] Handwerger S, Raucher B, Altarac D, Monka J, Marchione S, Singh KV, Murray BE, Wolff J, Walters B (1993). Nosocomial outbreak due to Enterococcus faecium highly resistant to vancomycin, penicillin, and gentamicin. Clin Infect Dis.

[B19] Karanfil LV, Murphy M, Josephson A, Gaynes R, Mandel L, Hill BC, Swenson JM (1992). A cluster of vancomycin-resistant Enterococcus faecium in an intensive care unit. Infect Control Hosp Epidemiol.

[B20] Morris JG, Shay DK, Hebden JN, McCarter RJ, Perdue BE, Jarvis W, Johnson JA, Dowling TC, Polish LB, Schwalbe RS (1995). Enterococci resistant to multiple antimicrobial agents, including vancomycin. Establishment of endemicity in a university medical center. Ann Intern Med.

[B21] Carmeli Y, Samore MH, Huskins C (1999). The association between antecedent vancomycin treatment and hospital-acquired vancomycin-resistant enterococci: a meta-analysis. Arch Intern Med.

[B22] Harbarth S, Cosgrove S, Carmeli Y (2002). Effects of antibiotics on nosocomial epidemiology of vancomycin-resistant enterococci. Antimicrob Agents Chemother.

[B23] Harris AD, Karchmer TB, Carmeli Y, Samore MH (2001). Methodological principles of case-control studies that analyzed risk factors for antibiotic resistance: a systematic review. Clin Infect Dis.

[B24] Ostrowsky BE, Venkataraman L, D'Agata EM, Gold HS, DeGirolami PC, Samore MH (1999). Vancomycin-resistant enterococci in intensive care units: high frequency of stool carriage during a non-outbreak period. Arch Intern Med.

[B25] Fridkin SK, Edwards JR, Courval JM, Hill H, Tenover FC, Lawton R, Gaynes RP, McGowan JE (2001). The effect of vancomycin and third-generation cephalosporins on prevalence of vancomycin-resistant enterococci in 126 U.S. adult intensive care units. Ann Intern Med.

[B26] Rubin LG, Tucci V, Cercenado E, Eliopoulos G, Isenberg HD (1992). Vancomycin-resistant Enterococcus faecium in hospitalized children. Infect Control Hosp Epidemiol.

[B27] Quale J, Landman D, Saurina G, Atwood E, DiTore V, Patel K (1996). Manipulation of a hospital antimicrobial formulary to control an outbreak of vancomycin-resistant enterococci. Clin Infect Dis.

[B28] Anglim AM, Klym B, Byers KE, Scheld WM, Farr BM (1997). Effect of a vancomycin restriction policy on ordering practices during an outbreak of vancomycin-resistant Enterococcus faecium. Arch Intern Med.

[B29] Morgan AS, Brennan PJ, Fishman NO (1997). Impact of a vancomycin restriction policy on use and cost of vancomycin and incidence of vancomycin-resistant Enterococcus. Ann Pharmacother.

[B30] Adachi W (1997). Experience with Vancomycin Education and Order Sheet to Limit Vancomycin Use. Hospital Pharmacy.

[B31] Lai KK, Kelley AL, Melvin ZS, Belliveau PP, Fontecchio SA (1998). Failure to eradicate vancomycin-resistant enterococci in a university hospital and the cost of barrier precautions. Infect Control Hosp Epidemiol.

[B32] Montecalvo MA, Jarvis WR, Uman J, Shay DK, Petrullo C, Rodney K, Gedris C, Horowitz HW, Wormser GP (1999). Infection-control measures reduce transmission of vancomycin-resistant enterococci in an endemic setting. Ann Intern Med.

[B33] Shaikh ZH, Osting CA, Hanna HA, Arbuckle RB, Tarr JJ, Raad (2002). Effectiveness of a multifaceted infection control policy in reducing vancomycin usage and vancomycin-resistant enterococci at a tertiary care cancer centre. J Hosp Infect.

[B34] Fridkin SK, Lawton R, Edwards JR, Tenover FC, McGowan JE, Gaynes RP (2002). Monitoring antimicrobial use and resistance: comparison with a national benchmark on reducing vancomycin use and vancomycin-resistant enterococci. Emerg Infect Dis.

[B35] Lautenbach E, LaRosa LA, Marr AM, Nachamkin I, Bilker WB, Fishman NO (2003). Changes in the prevalence of vancomycin-resistant enterococci in response to antimicrobial formulary interventions: impact of progressive restrictions on use of vancomycin and third-generation cephalosporins. Clin Infect Dis.

[B36] Guglielmo BJ, Dudas V, Maewal I, Young R, Hilts A, Villmann M, Gibbs L, Gropper M, Jacobs R (2005). Impact of a series of interventions in vancomycin prescribing on use and prevalence of vancomycin-resistant enterococci. Jt Comm J Qual Patient Saf.

[B37] Harris AD, Lautenbach E, Perencevich E (2005). A systematic review of quasi-experimental study designs in the fields of infection control and antibiotic resistance. Clin Infect Dis.

[B38] Nelson RR (1998). Selective isolation of vancomycin-resistant enterococci. J Hosp Infect.

[B39] (1995). Recommendations for preventing the spread of vancomycin resistance. Recommendations of the Hospital Infection Control Practices Advisory Committee (HICPAC). MMWR Recomm Rep.

[B40] Harris AD, Bradham DD, Baumgarten M, Zuckerman IH, Fink JC, Perencevich EN (2004). The use and interpretation of quasi-experimental studies in infectious diseases. Clin Infect Dis.

[B41] Wagner AK, Soumerai SB, Zhang F, Ross-Degnan D (2002). Segmented regression analysis of interrupted time series studies in medication use research. J Clin Pharm Ther.

[B42] Montecalvo MA, Jarvis WR, Uman J, Shay DK, Petrullo C, Horowitz HW, Wormser GP (2001). Costs and savings associated with infection control measures that reduced transmission of vancomycin-resistant enterococci in an endemic setting. Infect Control Hosp Epidemiol.

